# Supplementary material for: 18F-Fluorodeoxyglucose Positron Emission Tomography Is Useful in the Evaluation of Prognosis in Retroperitoneal Sarcoma
Source: Cancers (Basel). 2021 Sep 14;13(18):4611. doi: 10.3390/cancers13184611 (PMC8471941; doi:10.3390/cancers13184611)
Supplement: Supplementary file 1 [file cancers-13-04611-s001.zip › cancers-1356359-SI.pdf]

cancers

MDPI

Article

18F-Fluorodeoxyglucose Positron Emission Tomography Is Useful in the Evaluation of Prognosis in Retroperitoneal Sarcoma

Toru Wakamatsu [1,2,*], Yoshinori Imura [1,2], Hironari Tamiya [1,2], Toshinari Yagi [2], Naohiro Yasuda [1], Sho Nakai [1,2], Takaaki Nakai [3], Hidetatsu Outani [1], Kenichiro Hamada [1], Shigeki Kakunaga [3], Nobuhito Araki [2], Takafumi Ueda [3] and Satoshi Takenaka [1,2]

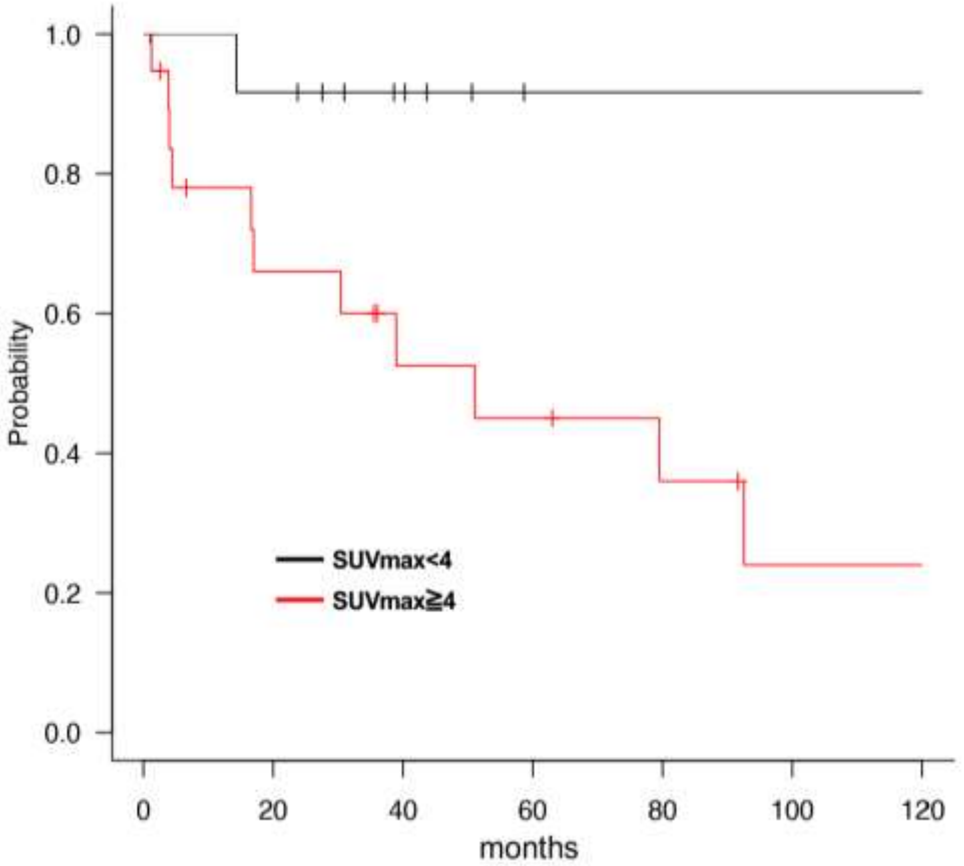

Figure S1. OS of RPS patients with SUVmax low (<4) or SUVmax high (≧4) in group of other.

Table S1. Analyses of survival in other patients.

			Univariate analysis		
**Variable**		**No.**	**5 year-OS (%)**	**95% CI (%)**	***P* value**
Age	60>	17	59.2	23.7-82.6	0.882
	60≦	28	60.1	35.6-77.9	
Gender	Male	24	37.8	15.8-59.9	0.0214
	Female	21	90	65.6-97.4	
Metastasis at first visit	Yes	8	NA	NA	<0.001
	No	37	66.8	44.2-82	
Tumor Grade	1	17	100	NA	0.00237
	2 and 3	23	29.9	10.8-51.9	
Tumor size	<5cm	10	87.5	38.7-98.1	0.0184
	≧5cm	34	51.3	28.9-69.8	
Surgical resection	Yes	26	66.6	37.4-84.5	0.0519
	No	19	49.2	22.2-71.6	
Chemotherapy	Yes	19	52.2	25.5-73.4	0.733
	No	26	67.6	36.7-85.8	
Radiotherapy	Yes	14	58.6	26.7-80.6	0.505
	No	31	61.5	34.5-80.1	
SUVmax	Low (4>)	13	91.7	53.9-98.8	0.0276
	High (4≦)	19	45	20.2-67.2	

Table S2. Multivariate Cox regression model of OS in other.

	multivariate analysis	
Covariates	HR (95% CI)	*P* value
Age (60> vs 60≦)	0.1522 (0.021251.091)	0.06103
Gender (Male vs Female)	0.1168 (0.01079-1.264)	0.07718
Metastasis at first visit (Yes vs No)	23.1 (1.216-438.8)	0.03663
Tumor Grade (1 vs 2 and 3)	2.071 (0.2277-18.84)	0.518
Tumor size (<5cm vs ≧5cm)	0.7958 (0.1009-6.276)	0.8284
Surgical resection (Yes or No)	0.1008 (0.01162-0.8741)	0.03733
Chemotherapy (Yes or No)	0.1908 (0.03451-1.055)	0.0576
Radiotherapy (Yes or No)	0.5015 (0.06777-3.711)	0.4992
SUVmax (4> vs 4≦)	16.47 (0.5837-464.9)	0.1002
